# Chromosomal Instability and Phosphoinositide Pathway Gene Signatures in Glioblastoma Multiforme

**DOI:** 10.1007/s12035-014-9034-9

**Published:** 2014-12-15

**Authors:** Mark G. Waugh

**Affiliations:** Lipid and Membrane Biology Group, Institute for Liver and Digestive Health, UCL, Royal Free Campus, Rowland Hill Street, London, NW3 2PF UK

**Keywords:** PI 4-kinase, Cancer, Glioblastoma, Gene copy number

## Abstract

Structural rearrangements of chromosome 10 are frequently observed in glioblastoma multiforme and over 80 % of tumour samples archived in the catalogue of somatic mutations in cancer database had gene copy number loss for *PI4K2A* which encodes phosphatidylinositol 4-kinase type IIalpha. *PI4K2A* loss of heterozygosity mirrored that of *PTEN*, another enzyme that regulates phosphoinositide levels and also *PIK3AP1*, *MINPP1*, *INPP5A* and *INPP5F*. These results indicated a reduction in copy number for a set of phosphoinositide signalling genes that co-localise to chromosome 10q. This analysis was extended to a panel of phosphoinositide pathway genes on other chromosomes and revealed a number of previously unreported associations with glioblastoma multiforme. Of particular note were highly penetrant copy number losses for a group of X-linked phosphoinositide phosphatase genes *OCRL*, *MTM1* and *MTMR8*; copy number amplifications for the chromosome 19 genes *PIP5K1C*, *AKT2* and *PIK3R2*, and also for the phospholipase C genes *PLCB1*, *PLCB4* and *PLCG1* on chromosome 20. These mutations are likely to affect signalling and trafficking functions dependent on the PI(4,5)P_2_, PI(3,4,5)P_3_ and PI(3,5)P_2_ lipids as well as the inositol phosphates IP_3_, IP_5_ and IP_6_. Analysis of flanking genes with functionally unrelated products indicated that chromosomal instability as opposed to a phosphoinositide-specific process underlay this pattern of copy number variation. This in silico study suggests that in glioblastoma multiforme, karyotypic changes have the potential to cause multiple abnormalities in sets of genes involved in phosphoinositide metabolism and this may be important for understanding drug resistance and phosphoinositide pathway redundancy in the advanced disease state.

## Introduction

Glioblastoma multiforme is the most common malignant brain tumour and accounts for the majority of deaths from this disease. A number of comprehensive recent publications have described the genetic changes associated with glioblastoma multiforme [1–13]. However, rather than assessing global genomic changes, this study concerns mutations likely to affect phosphoinositide metabolism and signalling. The initial focus herein is on phosphatidylinositol (PI) 4-kinase type IIα (PI4K2A), a 55 kDa constitutively membrane-associated enzyme which localises mainly to the *trans*-Golgi network (TGN) and late endosomes where it catalyses the formation of PI4P by D4 phosphorylation of its substrate lipid phosphatidylinositol [14]. PI4K2A is unique amongst the phosphoinositide kinases in that it is regulated by dual palmitoylation within its catalytic domain and by its targeting to cholesterol-rich membrane rafts where its activity is very sensitive to alterations to membrane sterol levels [15–22]. A number of intracellular trafficking roles have been described for this enzyme including the recruitment of the clathrin adapters AP-1 [23] and GGA [24] to TGN membranes and AP-3 to late endosomes [25]. More recent work has revealed the enzyme’s function in regulating Wnt receptor signalling [26, 27] and described its reciprocal regulation on endosomal membranes by the ubiquitin ligase itch [28]. In the central nervous system, PI4K2A is by far the most prevalent PI kinase activity and is most strongly expressed in cerebellar Purkinje cells and astrocytes [29]. Astrocytomas and subsequently glioblastoma multiforme usually develop from astrocytes, and this prompted us to investigate whether *PI4K2A* mutations are associated with this disease.

Several studies have demonstrated that PI4K2A controls the signalling and trafficking of cell surface receptors such as EGFR [30–32] and HER2 [33] which are known to stimulate oncogenic signalling. EGFR is often overexpressed in glioblastoma [12] and anti-EGFR therapies are a potential targeted therapy in this difficult-to-treat disease [4, 13, 34–40]. In addition to EGFR overexpression, loss of the tumour suppressor PTEN is a common feature in glioblastoma [1, 41–43]. PTEN is a phosphoinositide 5-phosphatase that in normal cells rapidly dephosphorylatesthe PI3K lipid product PI(3,4,5)P_3_ which is a potent stimulator of signalling via the Akt/mTOR pathway [44–47]. Deletion of *PTEN* results in sustained and elevated PI3K signalling and augmented cell proliferation [48]. Given that PI4KIIα has roles in regulating both EGFR and AKT signalling, an initial aim of this study was to ascertain if the *PI4K2A* gene is mutated in glioblastoma. Through analysis of publically accessible genomic data available via the COSMIC resource, we were able to establish that point mutations of *PI4K2A* were rare in glioblastoma but more than 80 % of samples from a cohort of 638 exhibited loss of *PI4K2A* heterozygosity; this level of copy number variation mirrored that of *PTEN* which also localises to chromosome 10p.

## Methods

### Genomic Analysis

The Catalogue of Somatic Mutations In Cancer (COSMIC) bioinformatics resource (http://www.sanger.ac.uk/cosmic) [49, 50] established and maintained by the Wellcome Trust Sanger Institute was used in order to identify mutations in the 638 individual tumour samples analysed by the Cancer Genome Atlas (TCGA). For these samples in the COSMIC database, gene copy number analysis was carried out using ASCAT (Allele-Specific Copy number Analysis of Tumours) algorithm software [51] available at http://heim.ifi.uio.no/bioinf/Projects/ASCAT. For this analysis, the average copy number for the genome is 1.90 and a reduction in total copy number to a value of 1.30 or less demarcates a loss, while a gain was set at copy number ≥ 3.

### Protein Expression Analysis

The Human Protein Atlas [52, 53] (www.proteinatlas.org) online resource was utilised to investigate the expression of candidate proteins identified from the genomic analyses in immunohistochemically stained control cerebral cortex tissue and glioblastoma patient samples. The immunohistochemical data included here derives only from antibodies that also detected the correct size protein band on Western blots.

### String Analysis

The String [54] Search Tool for the Retrieval of Interacting Genes/Proteins software (http://string-db.org) was used to visualise interactions and networks amongst the protein products from the phosphoinositide pathway genes identified as having the highest levels of copy number variation in glioblastoma.

## Results and Discussion

**Fig. 1:**
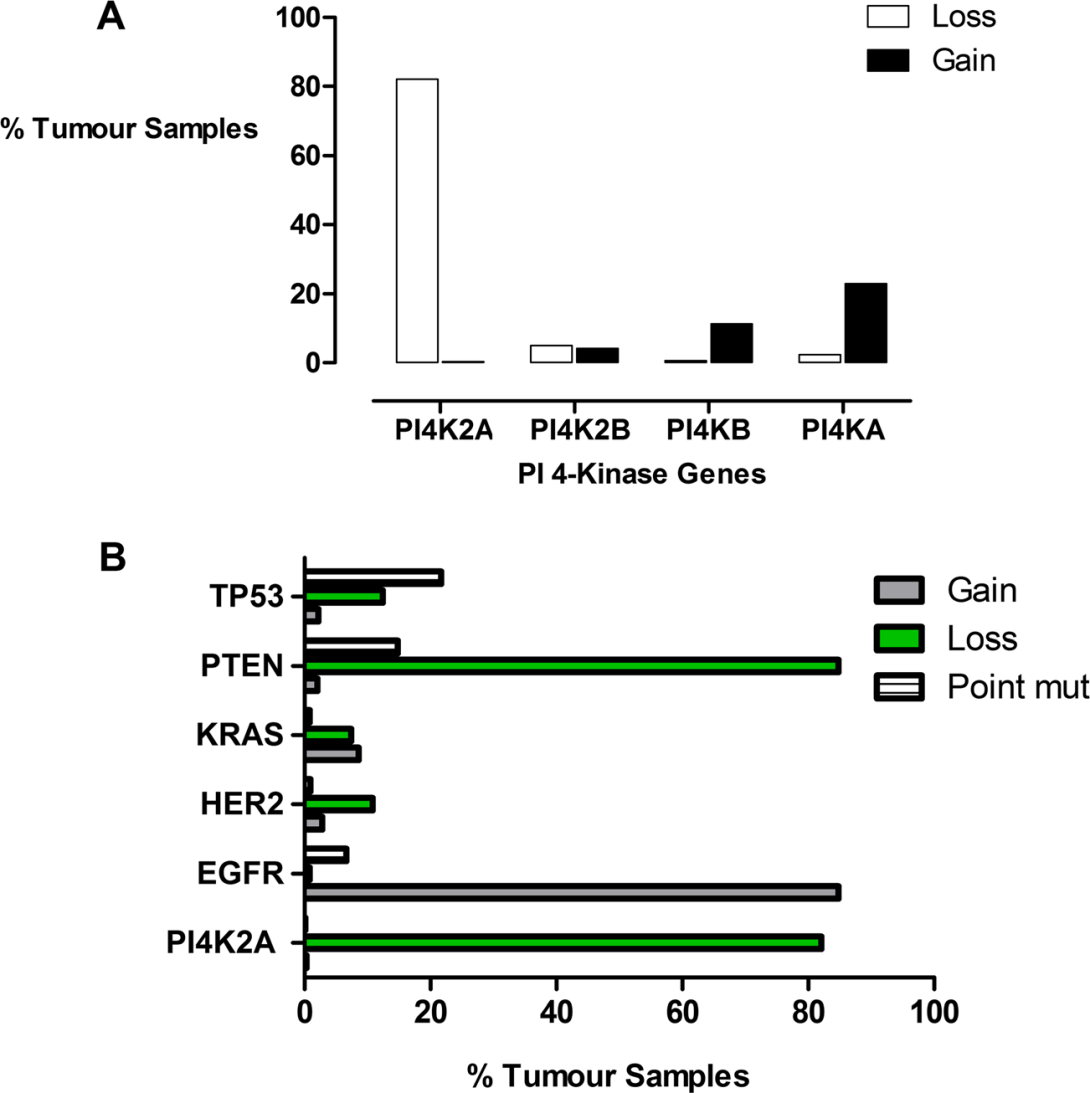
**a** Gene copy number variation for the four human PI 4-kinase isozymes in glioblastoma multiforme. **b** Comparison of gene copy number variation for PI4K2A with established oncogenes. Data was derived from mining the COSMIC database

As a first step, we sought to determine if there was any evidence for PI 4-kinase mutations in glioblastoma. We used the COSMIC resource and genomic data from 638 different glioblastoma patient samples to investigate the mutational status of the 4 human PI 4-kinase genes, *PI4KA*, *PI4KB*, *PI4K2A and PI4K2B*. Point mutations giving rise to amino acid changes were rare for all four PI 4-kinase genes and were observed in less than 1 % of all the samples in the combined patient cohort. However, analysis of gene copy number variation revealed that in 82 % of cases, there was a decrease in *PI4K2A* copy number and this was not seen for any of the other PI 4-kinase genes (Fig. 1). The pattern of *PI4K2A* copy number variation was also compared with a panel of established oncogenes and tumour suppressors composed of *PTEN*, *EGFR*, *HER2* and *KRAS* (Fig. 1). From this initial comparison, it became apparent that the level of *PI4K2A* gene copy number loss in glioblastoma mirrored that of a tumour suppressor such as *PTEN* as opposed to an oncogene such as *EGFR* that had an increased copy number. The loss of heterozgosity for *PTEN* and *PI4K2A* is biologically significant as these genes both localise to chromosome 10q and suggests that their copy number variation was due to a chromosome 10q deletion. For this to be the case, then the nearest neighbour genes to *PI4K2A* should also undergo a similar degree of gene copy number loss. To investigate this possibility, two genes immediately upstream and downstream of *PI4K2A* were identified. The upstream genes at the 10q24.2 locus were *HOGA1* and *MORN4* while the immediate downstream genes were *AVPI1* and *MARVELD1*. Consistent with a copy number change caused by chromosomal loss, the pattern of copy number variation (0.2 % gain and 82 % loss) for each of these flanking genes in glioblastoma was almost identical to *PI4K2A* and *PTEN*. However, unlike PTEN and PI4K2A, there is no evidence for any of these flanking gene products being involved in receptor stimulated oncogenic signalling. These results also demonstrate that the copy number reduction of genes localised to chromosome 10 was not specific to the phosphoinositide pathway and that these changes could be explained by an overall loss of chromosome 10 heterozygosity.

For a more complete analysis of genomic changes across the entire phosphoinositide pathway, a panel of genes encoding proteins involved in phosphoinositide metabolism, signalling and trafficking was investigated. Forty-five additional genes encoding phosphoinositide kinases, phosphatases, phospholipase C isozymes and also effector proteins such as the three Akt isoforms were included in this expanded analysis. Amongst the glioblastoma samples on COSMIC, copy number variation was frequently observed in this group of genes (Table 1). Copy number loss was most pronounced for phosphoinositide pathway genes localised to both arms of chromosome 10 and the X chromosome. For copy number amplifications, the greatest increases were seen for genes on chromosomes 19 and 20 followed by chromosomes 3 and 1. This rank ordering of chromosomes is in line with their known susceptibilities to undergo deletion or amplification in glioblastoma [42, 55–57]. Significantly, using this approach, it was possible to identify 17 new gene mutations, present in at least 10 % of the glioblastoma multiforme patient samples, with the potential to affect phosphoinositide metabolism and signalling.

**Table 1:** A summary of the copy number changes in glioblastoma multiforme for a panel of genes involved in phosphoinositide signalling and trafficking

Gene	Chromosome	Gain	Loss	Main effect on lipids	Previous report in glioma
INPP5B (1p34)	1	8.80	2.70	PI(4,5)P_2_ ↓	No
PIK3R3 (1p36.2)	1	8.80	2.20	PI(3,4,5)P_3_ ↑	Yes [88]
PI4KB (1q21)	1	11.30	0.60	PI4P ↑	No
PIP5K1A (1q21.3)	1	11.30	0.60	PI(4,5)P_2_ ↑	No
AKT3 (1q44)	1	12.20	2.00	–	Yes [89]
PIK3C2B (1q32)	1	19.80	0.60	PI(3,4)P_2_ ↑	Yes [45, 90, 91]
INPP1 (2q32)	2	2.50	3.80	IP_2_ ↑	No
INPP4A (2q11.2)	2	4.50	1.60	PI3P ↑	No
PIKFYVE (2q34)	2	3.50	2.40	PI5P/PI(3,5)P_2_ ↑	No
SACM1L (3p21.3)	3	5.80	7.80	PI4P ↑	No
PIK3R4 (3q22.1)	3	7.00	6.00	PI3P ↑	No
PIK3CB (3q22.3)	3	6.90	5.20	PI(3,4,5)P_3_ ↑	Yes [92]
PIK3CA (3q26.3)	3	11.80	3.50	PI(3,4,5)P_3_ ↑	Yes [93]
INPP4B (4q31.21)	4	3.60	5.80	PI(3,4)P_2_ ↑	No
PIK3R1 (5q13.1)	5	4.40	6.60	PI(3,4,5)P_3_ ↓	Yes [94]
FIG 4 (6q21)	6	1.60	18.80	PI(3,5)P_2_ ↑	No
MTMR7 (8p22)	8	6.30	8.00	PI3P ↑	No
PIP4K2A (10p12.2)	10	2.20	77.60	PI(4,5)P_2_ ↓	No
MINPP1 (10q23)	10	0.30	83.90	IP6 ↑	No
PTEN (10q23.3)	10	2.00	84.80	PI(3,4,5)P_3_ ↑	Yes
PIK3AP1 (10q24.1)	10	0.30	83.40	PI(3,4,5)P_3_ ↓	No
INPP5F (10q26.11)	10		84.30	PI(3,4,5)P_3_ ↑	No
INPP5A (10q26.3)	10		83.40	IP_3_ ↑	Yes [66]
PIK3C2A (11p15.5-p14)	11	2.50	12.10	PI3P/PI(3,4)P_2_ ↓	No
PLCB3 (11q13)	11	1.60	11.30	PI(4,5)P_2_ ↑	No
MTMR2 (11q22)	11	2.70	9.70	PI3P ↑	No
PIK3C2G (12p.12)	12	8.90	9.90	PI(3,4,5)P_3_ ↑ or ↓	Yes [95]
MTMR6 (13q12)	13	1.60	26.50	PI3P/PI(3,5)P_2_ ↑	No
AKT1 (14q32.32)	14	4.50	23.00	–	Yes
PLCB2 (15q15)	15	1.10	21.90	PI(4,5)P_2_ ↑	No
PLCG2 (16q24.1)	16	3.10	11.60	PI(4,5)P_2_ ↑	No
PIK3R5 (17p13.1)	17	2.70	10.70	PI(3,4,5)P_2_ ↑	No
PIP4K2B (17q12)	17	2.80	10.70	PI(4,5)P_2_ ↓	No
MTMR4 (17q22–q23)	17	6.40	5.50	PI3P ↑	No
PIK3C3 (18q12.3)	19	4.90	7.50	PI3P ↓	No
PIP5K1C (19p13.3)	19	30.20	5.00	PI(4,5)P_2_ ↑	No
PIP5K1B (19; 19 C1)	19	8.00	9.70	PI(4,5)P_2_ ↑	No
AKT2 (19q13.1–q13.2)	19	25.60	5.80	–	Yes [89, 96]
PIK3R2 (19q13.2–q13.4)	19	30.60	3.30	PI(3,4,5)P_3_ ↑	No
PLCB4 (20p12)	20	31.80	2.40	PI(4,5)P_2_ ↓	No
PLCB1 (20p12)	20	34.60	2.20	PI(4,5)P_2_ ↓	No
PLCG1 (20q12–q13.1)	20	30.30	2.20	PI(4,5)P_2_ ↓	Yes[97]
PIK3IP1 (22q12.2)	22	1.90	27.90	PI(3,4,5)P_3_ ↑	Yes [98]
MTMR8 (Xq11.2)	X	5.60	65.70	PI3P/PI(3,5)P_2_ ↑	No
OCRL (Xq25)	X	4.50	66.60	PI(4,5)P_2_ ↑	No
MTM1 (Xq28)	X	4.50	66.00	PI3P/PI(3,5)P_2_ ↑	No

The chromosomal localisation of each gene appears in brackets after its name. The “main effect on lipid” column refers to the predicted effect on either phosphoinositide lipid or inositol phosphate levels depending on whether gene copy number loss or gain predominates. The “previously reported in glioma” column indicates where there has been a previous publication demonstrating that a gene or gene product is involved in glioblastoma multiforme.

These results revealed that chromosomal instability affected particular sets of genes with the potential for chromosome-specific effects on phosphoinositide lipid levels metabolism (Table 1) with dyshomeostasis of PI(3,4,5)P_3_, PI(4,5)P_2_ and PI(3,5)P_2_ the most likely outcomes in glioblastoma multiforme. In the case of chromosome 10q loss, in addition to *PI4K2A* and *PTEN*, copy number losses in over 80 % of tumour samples were identified for four other phosphoinositide pathway genes. The effected genes were *PIK3AP1* (10q24.1) also known as B-cell adaptor for phosphatidylinositol 3-kinase (BCAP), *MINPP1* (10q23), *INPP5F* (10q26.11) and *INPP5A* (10q26.3). Two of these gene products, *PTEN* and *INPP5F,* are phosphoinositide 5-phosphatases and reductions in their enzymatic activities have the potential to induce sustained PI(3,4,5)P_3_ stimulation of the PI3K/mTOR pathway. However, unlike *PTEN*, *INPP5F* has not previously been considered as an oncogene in glioblastoma multiforme. Nevertheless, the potential decrease of PI3K lipid substrate brought about by the combination of *PI4K2A* loss and reduced PI3K activation due to diminished levels of the stimulatory protein PIK3AP1, could potentially off-set the effects of reduced PI(3,4,5)P_3_ degradation. Although this latter scenario is more speculative since there is no evidence yet for PI4K2A positively affecting PI(3,4,5)P_3_ generation [31] or for PIK3AP1 stimulating PI3K activity in the brain, even though this protein is a negative regulator of PI3K signalling and a potential tumour suppressor of cancers affecting other tissues [58–63].

Gene copy numbers for MINPP1 and INPP5A, two enzymes involved in the dephosphorylation of soluble inositol phosphates, were also reduced in the majority of glioblastoma multiforme patient samples. INPP5A [64–68] dephosphorylates the 5-position of the receptor-activated PLC product inositol (1,4,5)-*tris*-phosphate (IP_3_) which binds intracellular IP_3_ receptors to release Ca^2+^ stored in endoplasmic reticulum stores, while MINPP1 [69–72] is a 3-phosphatase that can hydrolyze multiple soluble inositol phosphate substrates including the downstream IP_3_ products inositol pentakisphosphate (IP_5_) and inositol hexakisphosphate (IP_6_). There is some evidence that IP_6_ can function asa possible alternative chemical energy supply to ATP with the potential to drive tumour growth under anaerobic conditions [71, 72]. In this way, loss of both chromosome 10 encoded inositol phosphatases has the potential to induce sustained and augmented signalling by receptors that activate phospholipase C. It is worth noting that with the exceptions of the established tumour suppressor *PTEN* and two reports mentioning *INPP5A* [64, 66], none of the other phosphoinositide pathway genes on chromosome 10 discussed here has hitherto been associated with glioblastoma multiforme.

Another genomic alteration of note identified here that affected 65 % of the glioblastoma multiforme patient samples was copy number loss for a set of three phosphoinositide phosphatase genes associated with the X chromosome. These genes were *MTMR8* (Xq11.2), *OCRL* (Xq25) and *MTM1* (Xq28), and once again none of these phosphoinositide genes has previously been associated with glioblastoma multiforme (Table 1). Loss-of-function mutations in the protein products of each of these genes are known to cause different X-linked neurological diseases [73, 74] and endosomal trafficking defects that feature accumulations of their respective substrate lipids; PI3P and PI(3,5)P_2_ in the case of MTM1 [75–77] and MTMR8 [78], and PI(4,5)P_2_ for OCRL [79]. This trio of phosphatase genes has not been previously linked to cancer but in light of their proposed functions in endosomal trafficking [80–82] and autophagy [78, 83, 84], it is possible that loss of this gene combination results in profound defects in the intracellular trafficking and degradation of receptor tyrosine kinases [75] and, consequently, augmented pro-tumourigenic and proliferative signalling.

A number of potential gain-of-function mutations due to chromosomal amplifications were also identified in the patient tumour samples and these included several phosphoinositide pathway genes never before linked to cancers affecting the central nervous system (Table 1). The main loci that exhibited copy number gain were chromosomes 20 and 19 in approximately 30 % of the glioblastoma samples, and chromosome 1 in around 10 % of the patient cohort. Interestingly, while chromosomal loss impacted heavily on phosphoinositide phosphatases, thereby potentially augmenting signalling outputs, gene copy number changes associated with chromosomal amplification were also found to be biased towards potentiating oncogenic signalling (Table 1). However, for gene amplifications, the main changes were not in phosphoinositide phosphatase genes but more so in genes encoding phosphoinositide kinases such as *PIP5K1C* (19p13.3) and *PIK3C2B* (1q32), second messenger-generating enzymes such as the PLC isozymes *PLCB4* (20p12), *PLCB1* (20p12) and *PLCG1* (20q12-q13.1) and phosphoinositide-dependent signalling proteins such as *AKT2* (19q13.1-q13.2). These findings imply that chromosomal numerical aberrations that result in either gain or loss of gene copy number in glioblastoma multiforme can result in pro-oncogenic signalling and trafficking defects via the phosphoinositide pathway. This raises the possibility these altered phosphoinositide signalling outputs could be selected for on the basis that they confer a survival advantage during tumour progression. Further functional studies are required to investigate this hypothesis.

**Fig. 2:**
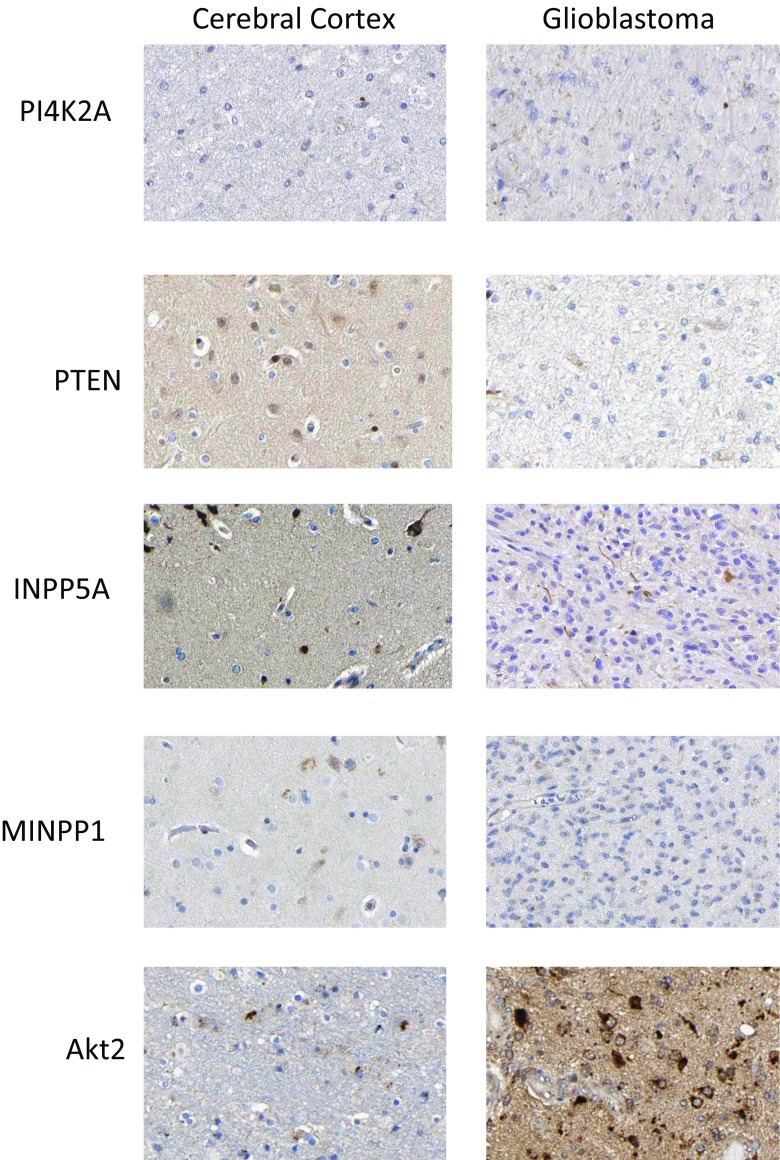
Expression of phosphoinositide pathway proteins in healthy cerebral cortex tissue and glioblastoma multiforme sections. Images were obtained by immunohistochemical staining of paraffin-embedded tissue samples using isoform-specific antibodies directed against the protein products of PI4K2A, PTEN, INPP5A, MINPP1 and AKT2. Antibody binding appears as brown/black staining on a background of blue haematoxylin counterstain. All images are from the Human Protein Atlas

The Human Protein Atlas archive of immunohistochemically stained cancer tissues was interrogated to uncover if the gene copy number variation in the phosphoinositide pathway was reflected in altered protein expression (Fig. 2). For comparison purposes, immunohistochemically stained normal cerebral cortex tissue was included in the analyses. However, the cytological composition of the cerebral cortex differs greatly from that found in glioblastoma tumour samples, with the former containing a high density of neurons whereas the latter is composed almost entirely of tumour cells derived from astrocytes. Thus, given these underlying histological differences, it is difficult to directly compare patterns of expressed proteins between control and patient samples. Notwithstanding these caveats, it was possible to identify high-grade glioblastoma samples that exhibited patterns of protein expression that were consistent with the gene copy number variation results. More specifically, high levels of gene copy number loss were found for *PTEN*, *PI4K2A*, *INPP5F* and *MINPP1* and there was concomitant reduced or absent expression of their cognate gene products in immunohistochemically stained glioblastoma tissues (Fig. 2). PI4K2A protein expression was not detected in 6/11 tumour samples; PTEN, was not detected in 10/12; INPP5A exhibited low to moderate staining in 3/12 diseased tissues and MINPP1 was not detected in 8/10 tumours. Conversely, for *AKT2* which was subject to a copy number increase, the corresponding protein products were intensely stained in high-grade glioblastoma samples. These images are included to provide some evidence that the protein expression patterns predicted by the genomic analysis could be observed in an independent immunohistochemical study. It is very important to note that these results derive from malignancies where the karyotype has not been characterised. Therefore it is not possible to conclude thatthe observed protein expression patterns are a consequence of chromosomal numerical abnormalities and associated changes in gene copy number. Proof of such a functional association is beyond the scope of the current in silico study, and future studies will aim to simultaneously investigate the link between glioblastoma karyotype, gene copy number status and the expression levels of enzymes involved in phosphoinositide signalling and trafficking.

**Fig. 3:**
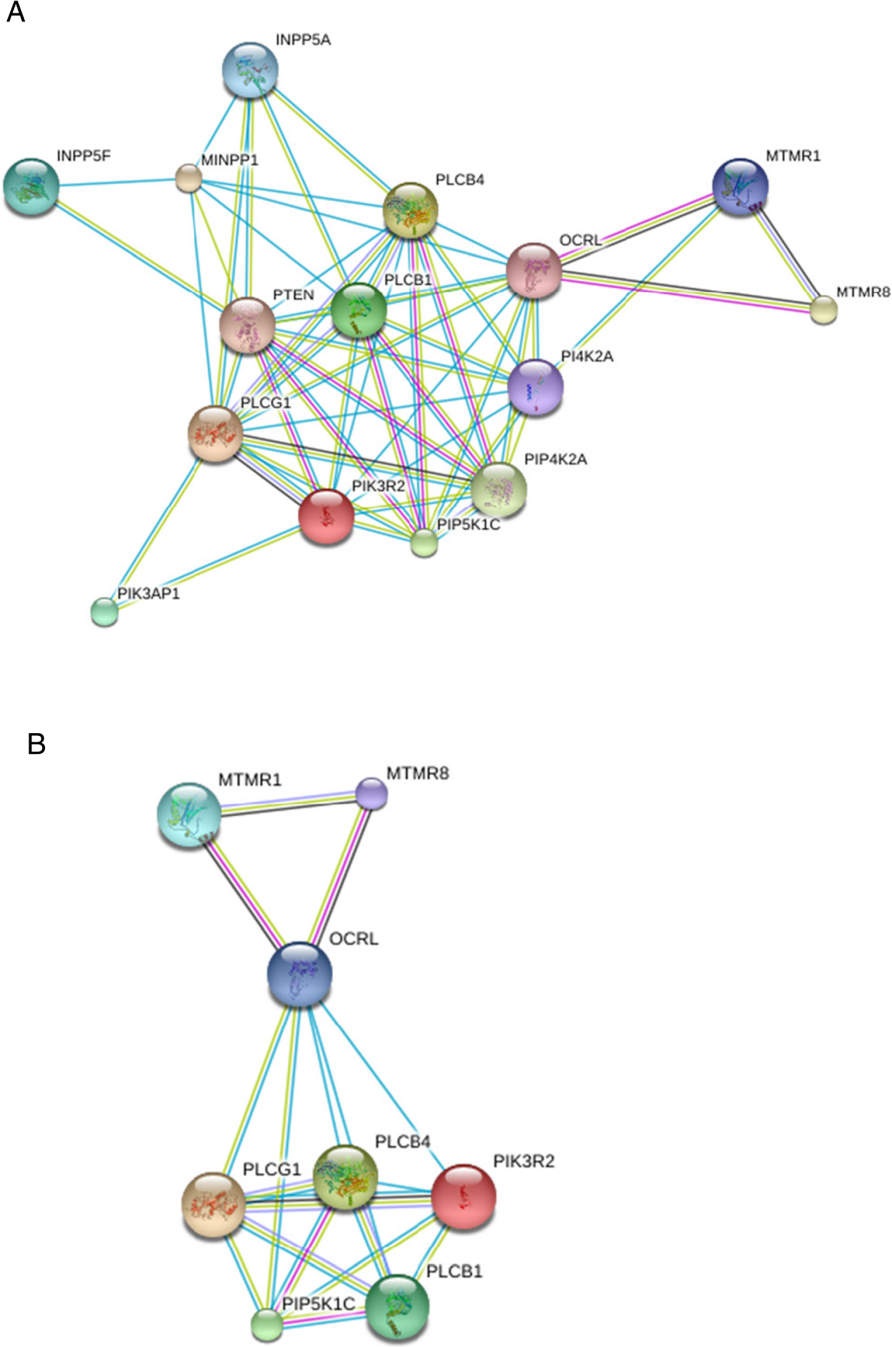
**a** STRING analysis illustrating the protein interaction network for the phosphoinositide pathway enzymes most commonly affected by gene copy number variation in glioblastoma multiforme. **b** Diagram illustrating how the protein interaction network would reset following a chromosome 10 deletion

The functional relationships between the most commonly affected phosphoinositide pathway gene products, defined as copy number variation in ≥30 % of samples, were assessed using freely available STRING software (Fig. 3). Using this approach it was established that all of the identified gene products are highly connected at the protein interaction level. Hence, a deletion or amplification of any one element in this putative pathway has the potential to reset the entire tumour-specific network of phosphoinositide-metabolising enzymes. To illustrate this scenario, STRING analysis is also shown for a tumour cell with a homozygous chromosome 10p deletion in order to show how the phosphoinositide metabolic network in glioblastoma could reorganise when a subset of gene products are lost through a gross karyotypic change. The majority of the affected gene products identified are involved in lipid dephosphorylation and hydrolysis, indicating that in glioblastoma, changes to phosphoinositide metabolism occur mainly via defective catabolism as opposed to altered synthesis. However, the degree to which the balance between synthesis and degradation is deregulated depends very much on the pattern of chromosomal instability in each tumour, thus leading to different permutations of phosphoinositide signalling and trafficking outputs contingent on which subset of genes is affected. These findings have implications for our understanding of drug resistance and target selection, particularly when it comes to chemotherapeutics aimed at inhibiting the PI3K/AKT/mTOR pathway [85–87]. Rather than focusing on a single enzyme, a more productive approach in cancer therapeutics could be to map the overall landscape of phosphoinositide pathway mutations present and to consider how these relate to the chromosomal structural abnormalities prevalent in aggressive cancers such as glioblastoma multiforme. This strategy will enable the identification of functionally redundant and alternative biochemical pathways to drug resistance that result from gene copy number variation and tumour-specific chromosomal aberrations.

## Abbreviations

IP_3_: Inositol(1,4,5)-*tris*-phosphate (IP_3_)
PI: Phosphatidylinositol
PI3K: Phosphoinositide 3-kinase
TGN: *trans*-Golgi network
